# Enhancing cancer treatment and understanding through clustering of gene responses to categorical stressors

**DOI:** 10.1038/s41598-023-33785-w

**Published:** 2023-04-21

**Authors:** Christopher El Hadi, George Hilal, Rita Aoun

**Affiliations:** 1 Faculty of Medicine, Saint-Joseph University, Beirut, Lebanon; 2 Cancer and Metabolism Laboratory, Faculty of Medicine, Saint-Joseph University, Beirut, Lebanon

**Keywords:** Cancer, Cancer genetics, Cancer genomics, Cancer metabolism, Cancer therapy, Tumour heterogeneity, Computational biology and bioinformatics, Classification and taxonomy, Computational models, Data acquisition, Data integration, Data mining, Data processing, Databases, Gene ontology, Genome informatics, Machine learning, Microarrays, Predictive medicine, Statistical methods

## Abstract

Cancer cells have a unique metabolic activity in the glycolysis pathway compared to normal cells, which allows them to maintain their growth and proliferation. Therefore, inhibition of glycolytic pathways may be a promising therapeutic approach for cancer treatment. In this novel study, we analyzed the genetic responses of cancer cells to stressors, particularly to drugs that target the glycolysis pathway. Gene expression data for experiments on different cancer cell types were extracted from the Gene Expression Omnibus and the expression fold change was then clustered after dimensionality reduction. We identified four groups of responses: the first and third were most affected by anti-glycolytic drugs, especially those acting on multiple pathways at once, and consisted mainly of squamous and mesenchymal tissues, showing higher mitotic inhibition and apoptosis. The second and fourth groups were relatively unaffected by treatment, comprising mainly gynecologic and hormone-sensitive groups, succumbing least to glycolysis inhibitors. Hexokinase-targeted drugs mainly showed this blunted effect on cancer cells. This study highlights the importance of analyzing the molecular states of cancer cells to identify potential targets for personalized cancer therapies and to improve our understanding of the disease.

## Introduction

In the 1920s, Otto Warburg and his colleagues observed a notable uptake of glucose in tumors in comparison to that observed in the surrounding tissues. Subsequent investigation revealed glucose fermentation, resulting in lactate production even in the presence of oxygen, a phenomenon termed "aerobic glycolysis"^1,2^. This paradoxical prevalence of glycolysis in cancer cells is referred to as the "Warburg effect." The catabolism of glucose to lactate yields a relatively low amount of energy, thereby necessitating high rates of glucose consumption to meet the energetic and anabolic demands of cancer cells^3^. In addition to providing energy, glycolysis generates metabolic intermediates that enable de novo synthesis of nucleotides, amino acids, lipids, and NADPH, all of which are essential for rapid cell proliferation^4^. Cancer cells thus possess a distinct metabolic profile that permits a high rate of proliferation and resistance to apoptosis signals^5^.

Inhibition of glycolytic pathways could be a promising selective approach in cancer research for developing targeted anticancer agents. Chen^6^ and Abdel-Wahab^7^ and colleagues have evaluated various inhibitory drug modalities that forestall glucose utilization. These drugs have been tested on cancer cells, followed by gene expression evaluation to identify more potent agents with a lethal metabolic impact on cancer cells. Currently, many glycolytic inhibitors are undergoing preclinical and clinical studies, demonstrating promising results^6,8,9^.

While many studies have described the effects of glycolysis inhibitory drugs on various cancers, there has been no effort to identify a common thread among the outcomes of these treatments. This study aims to fill that gap by examining how cancer cells respond to stressful environments, specifically those induced by glycolysis inhibitory drugs. By making use of fundamental cancer characteristics such as the molecules the drugs target, along with cancer “iClusters” extracted using an integrative clustering technique proposed by Shen et al*.*^10,11^, we hope to uncover hidden patterns that summarize cancer cell behavior under such conditions. The iClusters represent a new way of classifying all cancers based on molecular signatures rather than histology^12^. In their 2018 publication, Hoadly et al*.* expanded on this concept by analyzing approximately 33 tumor types or 10,000 samples and identifying 28 distinct iClusters using various types of data, including chromosome arm-level aneuploidy, DNA methylation, mRNA and miRNA expression, and protein expression^13^. With the insights gained from this study, we can hopefully make strides toward developing more effective cancer treatments.

## Results

**Figure 1:**
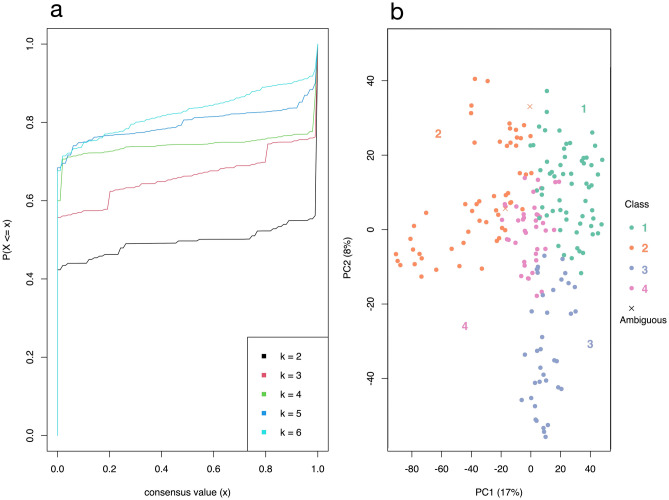
eCDF curve and PCA plot of the consensus clustering. (**a**) eCDF curve of the consensus matrix from partition by ATC:skmeans, a good k can be selected by aiming at the flatness of the eCDF curve. (**b**) PCA on 5000 rows with the highest ATC scores, 193 out of 195 confident samples were kept in their classes (silhouette > 0.5).

Two matrices were constructed, the first containing 199 experiments and the second 666. The rows amount to 33,622, representing the genes studied as expression fold-changes. The *adjust_matrix()* function of the *cola* package was used to clean the matrices. In the main matrix of 199 columns, 4 treatments were removed because more than 80% of their values were missing, leaving us with 195 samples and 16,127 genes filtered for analysis. Consensus partitioning recommended grouping the experiments into 5 classes, and alternatively into 2 or 4. Table 1 gives the evaluation indices for all classes with a maximum of 6, namely 1 − PAC (Proportion of Ambiguous Clustering), concordance, and mean silhouette. After calculating the product of 1 − PAC, concordance, and silhouette for the suggested three k, k = 4 classes was the highest k with a product ≥ 0.90 and was therefore preferred. Cumulative distribution function (CDF) curves for a maximum k of 6 and the PCA (Principal Component Analysis) plot for k = 4 are shown in Fig. 1a,b. Only treatments with silhouette scores ≥ 0.5 were retained in their corresponding classes, giving a new total of 193 analyzable experiments.

**Table 1:** Clustering evaluation indices for a maximum k of 6.

k	1-PAC	Mean silhouette	Concordance
2	0.989	0.970	0.987
3	0.870	0.907	0.958
4	1.000	0.963	0.980
5	0.949	0.888	0.949
6	*0.851*	*0.802*	*0.887*

*Abbreviations: PAC,* proportion of ambiguous clustering.

**Figure 2:**
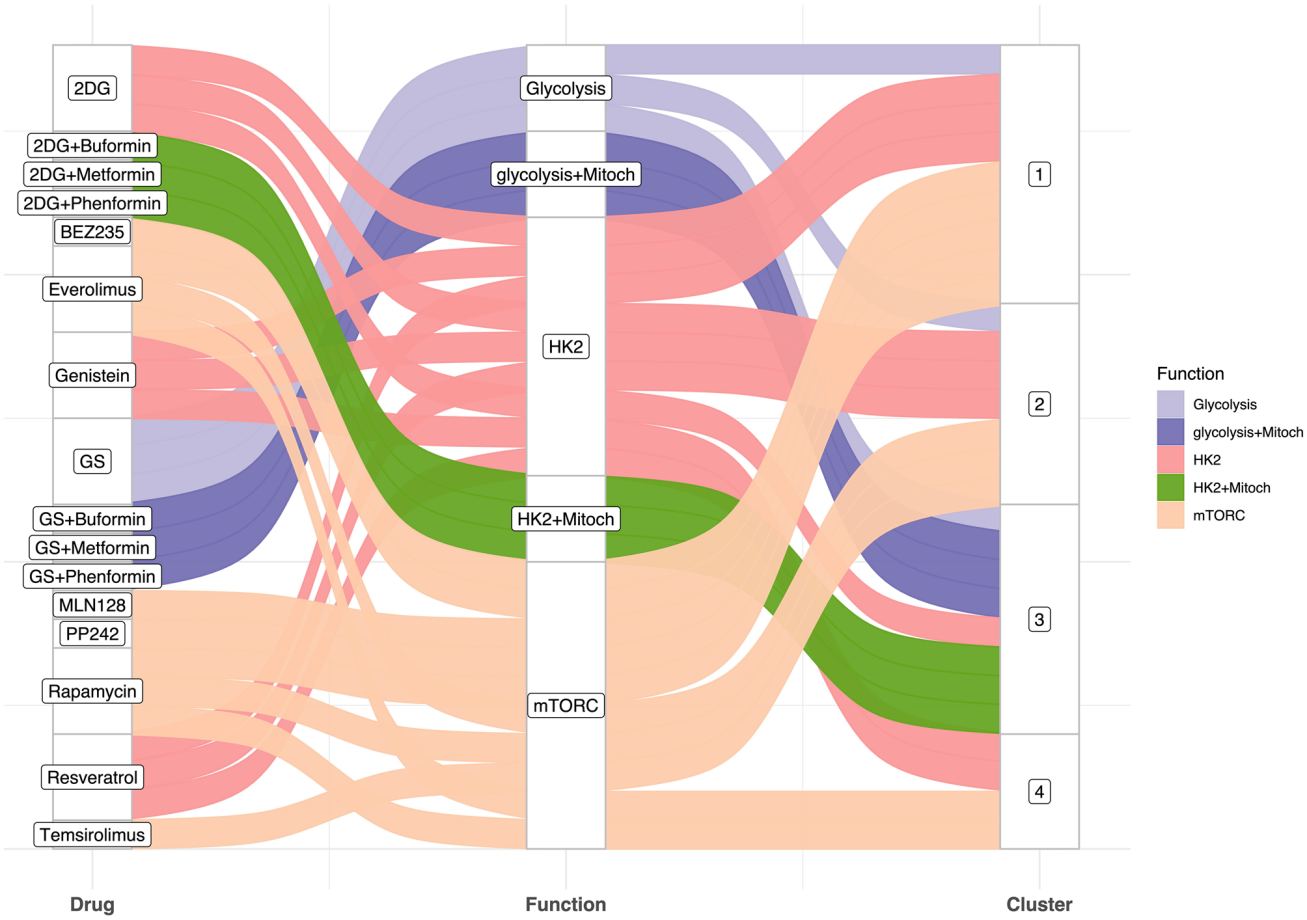
Relationship between the drugs studied, their molecular activity, and response clusters. The Sankey diagram shows molecular activity of each drug. Cluster designations are shown on the right.

The samples were not very different in their distribution between the classes (p = 0.054), with class 1 having 61 experiments, class 2 having 54, class 3 having 38, and class 4 having 40. Treatments were then classified by molecular mechanism and the goodness-of-fit test was applied to the frequency of experiments in each class. Table 2 shows the treatments used and their respective molecular mechanism as explained by Abdel-Wahab. Treatments acting on Hexokinase 2 (HK2) showed a significant preference for class 2, and those acting on mTORC variants (1/2) showed a high affinity for class 1. The combination of treatments acting on mitochondria (i.e., biguanides) with those acting on HK2 or with glucose restriction, constituted a type of "pan-glycolysis" inhibition and had a preference for class 3. This classification in class 3 was also observed with glucose deprivation, a true "pan-glycolysis" inhibition, which also showed an affinity for class 1. Nevertheless, it should be noted that drugs targeting mitochondrial function and the KRAS pathway, alone, did not show a predilection for any class, demonstrating their broad range of activity. The results described are visualized in Fig. 2.

**Table 2:** Actions of studied drugs on the glycolytic pathway.

Action	Drug	Action	Drug
Akt	Afuresertib	Mitochondria	Buformin
GLUT	Silybin		Metformin
Glucose	No Glucose		Phenformin
HK2	2DG	mTORC	Everolimus
	Genistein		MLN128
	Resveratrol		PP242
KRAS mutations	Selumetinib		BEZ235
	Sorafenib		Rapamycin
	Trametinib		Temsirolimus
		PDK	DCA

**Figure 3:**
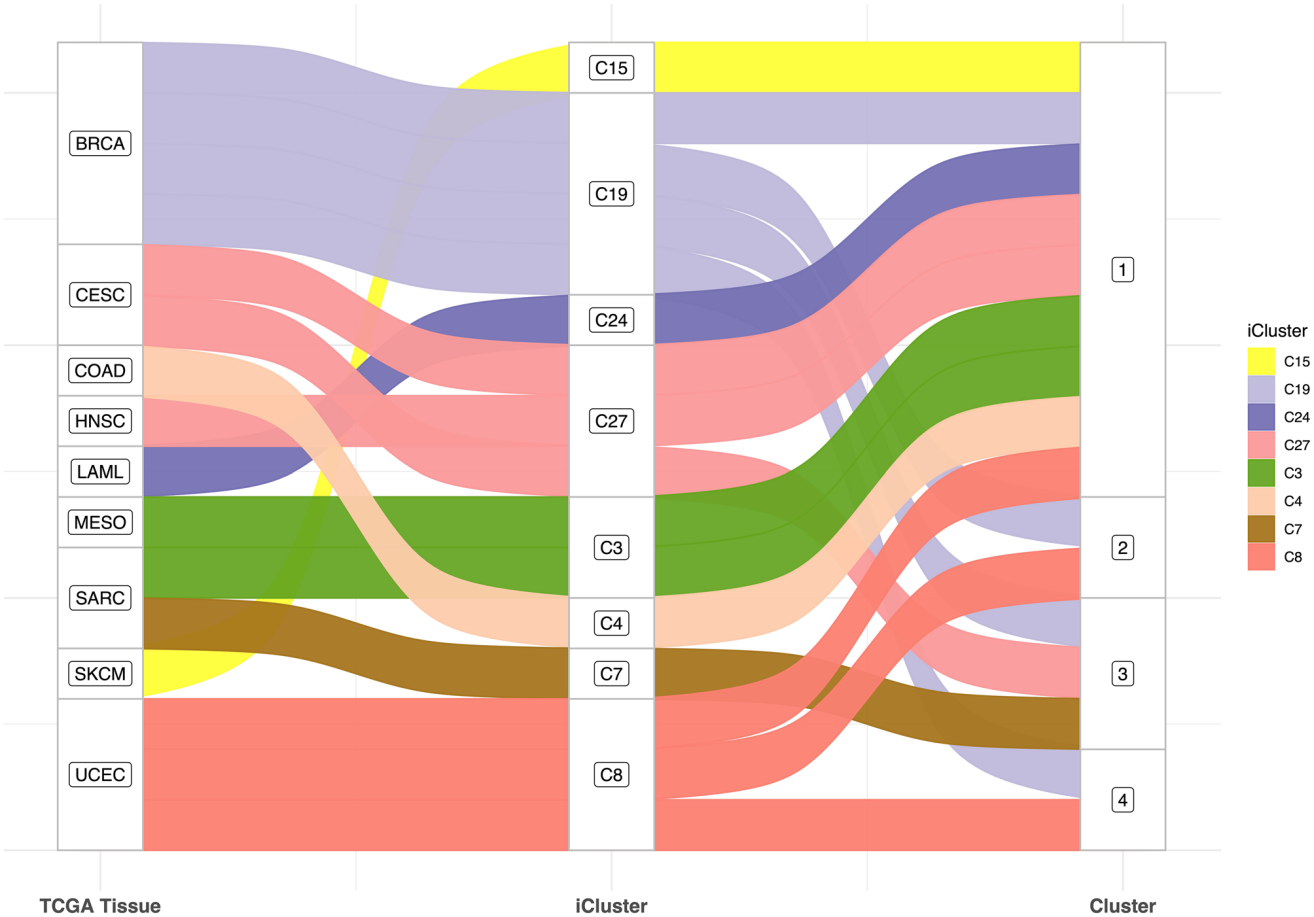
Relationship between TCGA tumor type, iCluster, and response clusters. The Sankey diagram shows the tumor type composition of each iCluster. Cluster designations are shown on the right.

In terms of cell type, Cluster 1 appears to harbor the majority of cells, comprising iClusters 15 (C15) and C24, these exclusively and respectively comprising SKCM (skin/new melanoma) and LAML (myeloid leukemia) cells. C15 is known to be affected by UVB exposure and of having the highest median mutation per megabase, and C24 is known to have the lowest median mutation per megabase among all iClusters. Similarly, C10, C25, and C27 (partitioning all squamous cell cancers) were predominantly classified in group 1 alongside group 3, as were C22, C7, and C3, enriched in SARC (sarcomas, primarily bone). One cell type predominates in C10 and C27, namely lung and cervical squamous cell cancers, the latter being HPV-related. Moreover, C10, C25, and C27 are known to be enriched with squamous-cell and proliferation-related pathways, high hypoxia levels, immune-related signaling, and basal signaling. Of note, C25 is specifically enriched with chr11 amplification. As for C22, C7, and C3, these iClusters were predominantly mesenchymal, most commonly having a chr9 deletion, and were enriched in gene programs representing PD1, CTLA4, and JAK2/STAT1, 3, 6 signalings. Cluster 2 was primarily enriched in C8 and C19, themselves known to cluster BRCA and UCEC (breast and uterus), with C8 also found almost exclusively in cluster 4. C8 is also a homogeneous iCluster, dominated by a single tumor type, with the POLE mutation present in highly somatically mutated cells. Together with C19, they both demonstrate hormone and cell of origin dependence, with C19 furthermore expressing the estrogen signaling gene program. Figure 3 summarizes this repartitioning and Supplementary file S3 illustrates the analysis process described for the drugs and tissue group distribution.

**Figure 4:**
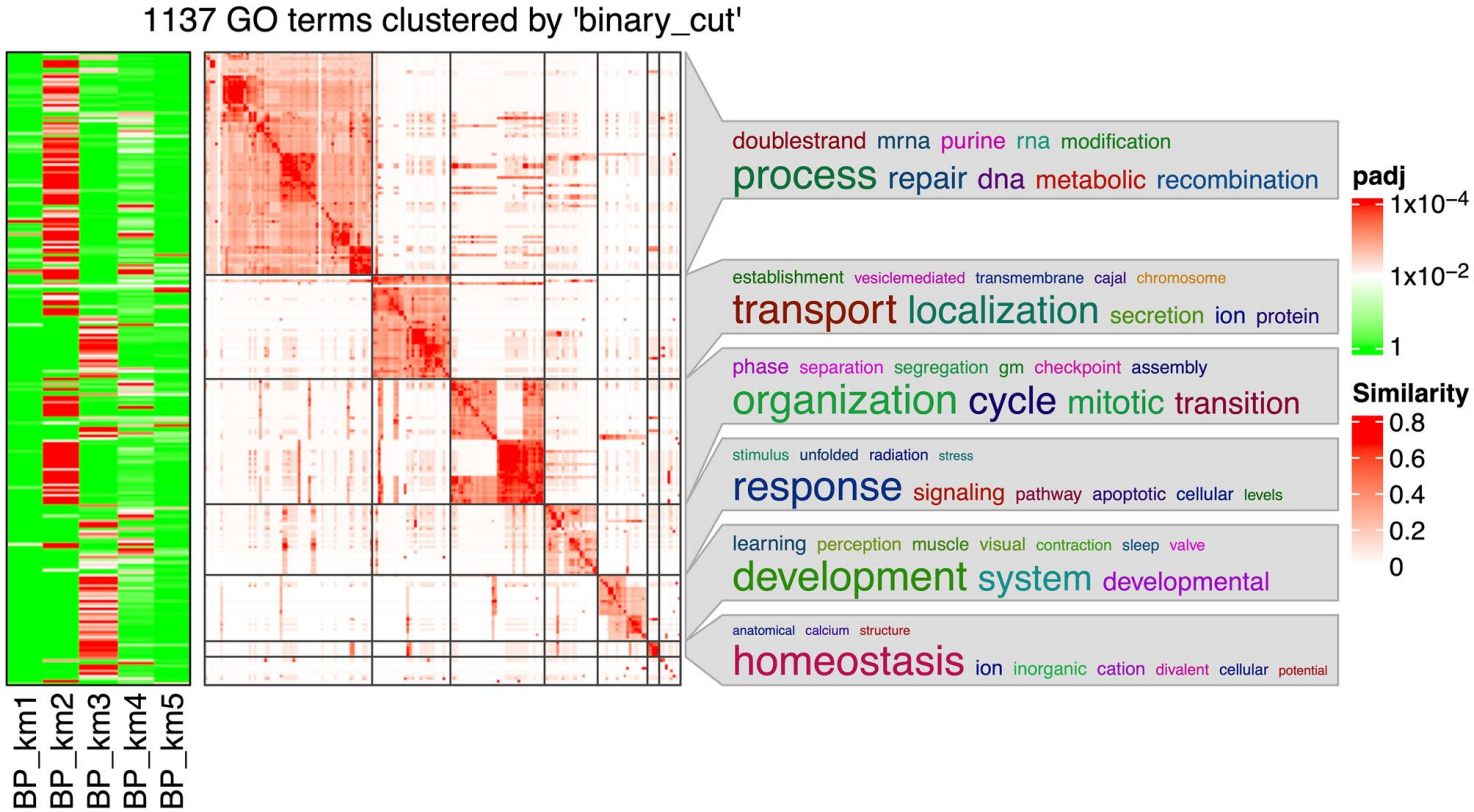
Heatmap of the GO term similarities from the 195-sample gene list. The terms resulted from the enrichment of the genes in either group (using FDR < 0.01). The green–red columns show for which group the respective GO terms are significant. The word cloud keywords highlight the biological functions in each GO group.

**Figure 5:**
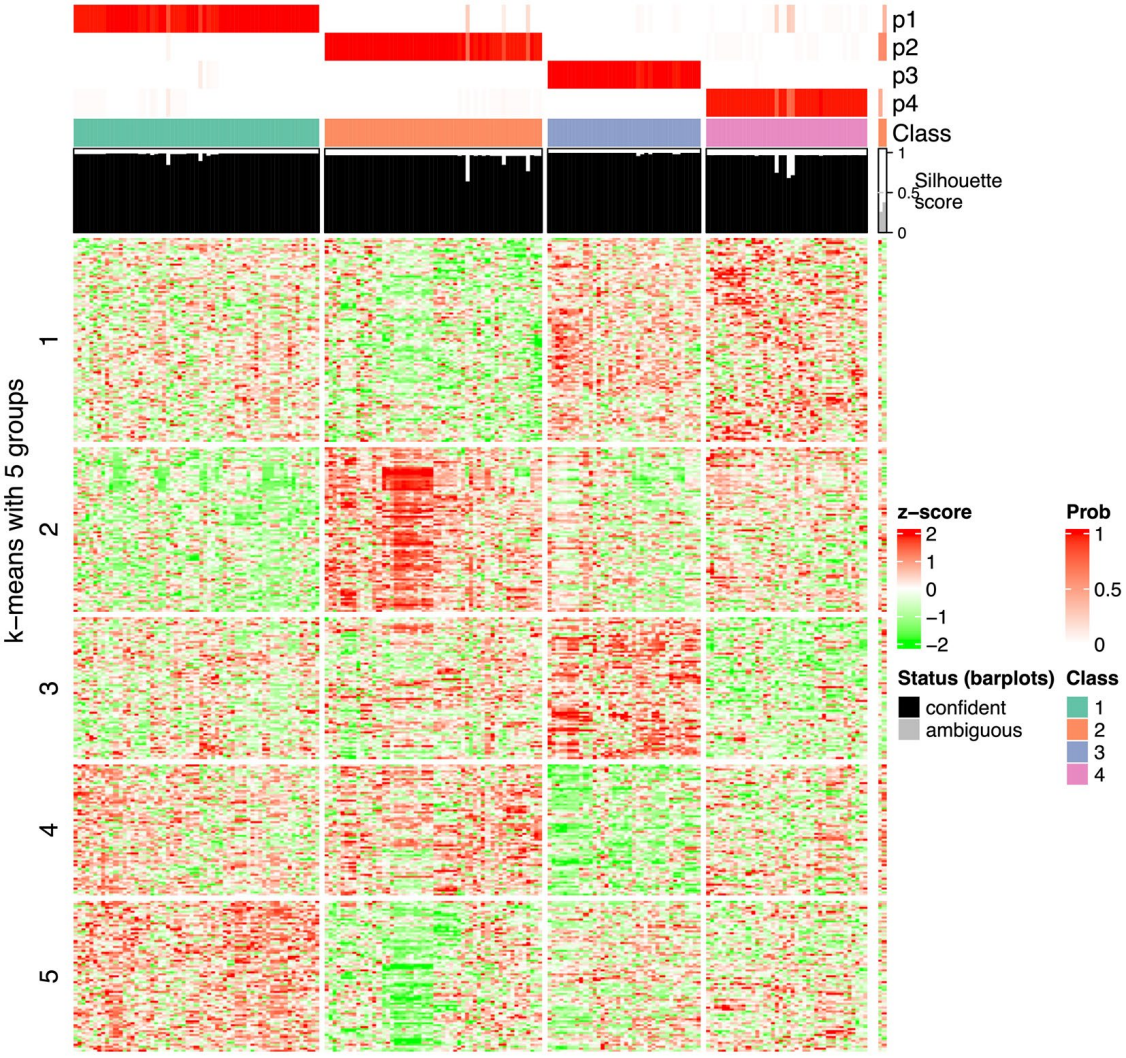
Heatmap of the signature genes and their regulation in each class. 9368 signature genes (58.1% of total genes) chosen for an FDR < 0.01. This heat map represents the differences in expression relative to the average FC for each gene. Table 3. Summarizes these differences.

Signature genes were identified using a false discovery rate (FDR) threshold of < 0.001. Clustering of genes by K-means yielded 5 signature functions, 2 of which were biologically more meaningful than the others. Functional analysis of the first function group using Gene Ontology (GO) terms for biological properties resulted in 17 term clusters (Fig. 4). Function A addresses cell cycle inhibition, including (i) DNA replication and repair, as well as RNA splicing and telomere maintenance, and (ii) mitotic cell cycle and regulation. Function B addresses cancer adaptation and molecular quality control mechanisms describing (i) apoptosis and the unfolded protein response (UPR) and related aspects of (ii) RNA and protein transport and localization in cells, (iii) organ development (perception and learning), and (iv) ion homeostasis. The activity of these functions in each group relative to the others (maximum activity, minimum activity, or baseline activity) is summarized in Table 3 and can be visualized in Fig. 5.

**Table 3:** Function groups and their activity in each cluster.

Cluster/function	A: Mitotic activity and DNA repair	B: Quality control, UPR and apoptosis
1	↓	–
2	↑	–
3	↓	↑
4	–	↓

Abbreviations: ↓ minimum activity; ↑ maximum activity; – baseline or reference activity; *UPR,* unfolded protein response.

The previously listed functions describe the expression of biological pathways that are different between classes, regardless of whether they are up or down-regulated in each experiment (significantly high FC). That said, up- or down-regulated responses remain to be explored.

Because our data were quantile-normalized, an FC ≥ 0.5 or ≤ − 0.5 was considered to define an up- or downregulated gene, respectively. Only classes 1 and 3 could be enriched, showing downregulation in mitotic activity and DNA replication, and class 3 showed additional upregulation in apoptotic activity and cellular stress response, cellular differentiation, and immune activation of cells.

Additional consensus clustering was performed on 341 sorafenib-treated and 325 rapamycin-treated samples (from the accession of 1014 samples), with each treatment having primarily 3 available doses given for 3 exposure times. Consensus clustering of rapamycin samples resulted in 3 classes and 4 classes of sorafenib. This classification takes into account the drug used and leaves room for classification and interpretation based on tissue, dose, and time. The classes obtained for both treatments simply split the samples almost evenly between them, which is true for tissue, treatment duration, and dose if tissue-dose and tissue-duration interactions were considered. Therefore, no hidden trends or categorization of these parameters could be inferred. It should be noted, however, that when tissue and treatment duration or dose interactions were not considered, there was a preference for certain duration and time classes (P < 0.01). Treatment signature genes were also classified and resulted in the same biological processes as described above, with organ development/differentiation being more vascular.

It was deemed unnecessary to combine the 666 experiments with the 195 and perform a clustering analysis because of the potential bias that could result from adding more than 300 samples treated with only 2 agents, to 195 samples treated with a total of 26 agents. In addition, partitioning that holds the tissue variable constant and allows for a treatment-based study was also deemed inefficient due to the limited number of samples per tissue.

## Discussion

This study used Gene Expression Omnibus (GEO) gene expression data to cluster cancer gene responses to stressors, particularly drugs that inhibit the glycolysis pathway. Unsupervised clustering after dimensionality reduction revealed four groups of responses, which exhibited distinct molecular characteristics and sensitivity to different drug classes.

Our results first highlight the importance of "pan-glycolytic" targets, in particular glucose restriction, which showed the best anticancer effect among all other treatments. This pan-glycolytic effect can be achieved by combining compounds, the most potent being those that simultaneously affect the cytoplasmic and mitochondrial phases of glucose oxidation. The only effective single-agent therapies are those that target mTOR variants which, unsurprisingly, play an important role in cancer growth and metabolism^14^, underscoring the importance of multi-targeted therapies to target cancer.

Second, it has become clear that patients with mesenchymal and squamous tumors can benefit from these pan-glycolytic/-like targets. Moreover, drugs such as biguanides and those targeting KRAS mutations may be considered as well. However, patients with hormone-sensitive tumors are unlikely to benefit from any anti-glycolytic drugs but may consider a trial of pan-glycolytic therapies and drugs acting on mitochondrial proteins and KRAS mutations. It should be noted that hormone-sensitive cells are considered to be well adapted to hypoglycemic environments and glycolysis challenges.

Our results also showed that clusters 1 and 3 had significant gene regulation that is worth elaborating upon. On the first hand, tumors classified in these clusters showed downregulation in mitosis, DNA replication, and all associated pathways and quality control checkpoints. This should mean that the drugs induced Warburgian stress that disrupted normal cell cycle processes, potentially slowing down or halting cell growth and division. On the other hand, cluster 3 got enriched with immune-related gene expression; knowing that cell cultures lack immune cells from their microenvironment, this expression can be due to various factors, including autocrine immune-related signaling^15^, stress-induced signaling and death associated with exposure and production of damage-associated molecular patterns (DAMPs)^16^, and possible persistence of immune cell from the original tissue. The additional detection of genes related to development and differentiation can be due to the initiation of adaptative responses to the stressful environment by evolutionary favoring of more resistant mutants through, for instance, incomplete epithelial–mesenchymal transition (EMT) or mesenchymal–epithelial transition (MET)^17,18^, and the ability of cancer cells to dedifferentiate or transdifferentiate into different cell types^19^.

All of the studies we reviewed for data interrogation, which investigated the response of specific cell lines to a single dose and duration of exposure of an antiglycolytic drug, demonstrated similar biological functions to ours (GEO accession numbers of the studies that demonstrated deregulation of class 1 and 3: GSE31058, GSE97346, GSE59882, GSE79316, GSE73923, GSE59228, GSE36847, GSE25412, GSE114060, GSE96794, GSE79246, GSE9008, GSE62663, GSE116387, GSE137553, GSE112079; and class 2 and 4 dysregulation: GSE59704, GSE5200, GSE85257, GSE112079. See Supplementary Table S1).

The distribution of cancer cells and drugs is not binary and must be taken into account when interpreting the results due to the complex and diverse nature of the experiments. The differences are attributed primarily to the heterogeneity of the experiments, acting on the unique metabolism of each of the 79 cell lines tested by exposing them to different doses and durations of 26 reagents. The different levels of activity of biological pathways in each cell are due to developmental and differentiation programs, and epigenetic states of the cells of origin in conjunction with exogenous factors, such as mutagenic exposures, pathogens, and inflammation. This explanation should be extended to the cancer microenvironment when translating the results of cancer responses to in vivo studies. The cancer microenvironment, which includes neighboring cells, blood vessels, and the extracellular matrix, can affect the expression of glycolysis-related genes^20,21^, limit drug delivery to tumor cells, and alter the immune response to the tumor^22,23^.

Asking for more in vitro experiments to get more statistically significant results is theoretically advantageous, but practically useless knowing that we are asking for more combinations of experiments that take us away from the intent of any analysis of this kind. We believe that there must be a hidden pattern, imperceptible to the human brain, that connects human cancers by allowing them to be positioned on a categorizable continuum. This explains why we used iClusters instead of tissues to categorize cells and explore what lies beyond simplistic tissue-based classifications^24^.

One limitation of our study is that we exclusively utilized microarray gene expression profiling as the analytical tool to investigate the genetic responses of cancer cells to anti-glycolytic agents. Despite the potential of microarray studies to offer valuable insights into gene expression alterations, they may not comprehensively capture the entire spectrum of genetic variations that manifest in cancer cells, including epigenetic changes and mutations. Thus, the findings of our study may not completely portray the intricate molecular landscape of cancer cells and their reaction to stressors. Forthcoming in silico investigations employing supplementary methodologies, such as single-cell sequencing and whole-genome sequencing, and quantifying responses within a cell by detecting genomic, proteomic, and other omics modules so as to compare these biological modules across cells, could furnish deeper knowledge into the molecular states of cancer cells and their response to stressors.

## Conclusions

While several studies have examined the effects of a single antiglycolytic drug on specific cell lines, none, to our knowledge, have used response clustering to analyze cancer. Our study aimed to fill this gap by identifying categories of cancer cell responses to a wide range of antiglycolytic agents. This classification can provide insight into the expected effects of any drug targeting glycolysis, helping to predict the response of cancer cells to such drugs.

Our results revealed unique molecular characteristics and varying levels of sensitivity to different drug classes, highlighting the importance of multi-targeted therapies in cancer treatment. Patients with mesenchymal and squamous tumors may benefit from mTOR and pan-glycolytic targets, whereas those with hormone-sensitive tumors are unlikely to benefit from anti-glycolytic drugs. Finally, it is important to consider the cancer microenvironment when applying these findings to in vivo studies.

## Methods

### Data processing

Gene expression profiles were downloaded from the GEO (Gene Expression Omnibus) database. The terms entered in the search engine were “Cancer” followed by the name of the agent, or any of the alternative names for the same agent. All the dataset accession numbers and their respective drugs and cell lines can be found in Supplementary Table S1. The raw data were also provided in Supplementary Tables S3 and S4. The filters used with every search were “Homo sapiens” for organisms, “Expression profiling by array” for the study type, and “Series” under the entry type. The inclusion criteria we based our judgment on for choosing the datasets were:

1. Expression data should only be from single-channel microarray chips. Agilent, Affymetrix, and Illumina are the only accepted manufacturers.
2. Cells should be treated in an artificial in vitro milieu (no mice xenografts etc.).
3. Cells should not have been transfected or mutated to affect the targeted pathway or been made resistant to treatment (only wild cell types under the influence of chemicals).
4. Cells should not have undergone secondary treatments (other than the vehicle and the drug of interest) before RNA extraction.
5. Coding RNA, particularly cytoplasmic or total extractions, is preferred.
6. The glucose deprivation medium should contain less than 1 g/L, i.e., 5.6 mM, of glucose.
7. Cells should be cancerous, not benign, with no other superimposed disease.
8. The data series should contain both controls and treatments.

The raw files downloaded were either in CEL or TXT format. Few TXT files contained processed data, and these were simply used as-is. For the Illumina Beadchips, R’s *limma*^25,26^ package was used and the function *neqc()* was employed to perform background correction followed by quantile normalization. It should be noted that negative control probes were not always available, therefore the detection p-values of each probe were exploited instead. For probe annotation, the *annotate* package was chosen^27^, with *illuminaHumanv3*.*db* as the annotation data^28^, and “gene symbol” as the annotation. For Affymetrix chips, *limma*, *affy*^29^, and *oligo*^30^ were used to process the CEL files. The *rma()* function normalized the microarray signals, and the annotation data packages were adapted to the version of Affymetrix chips available^31–38^. The Agilent chips were read using likewise the *limma* package, annotated according to the chip versions^39–41^, background-corrected following the normal-exponential method, and quantile normalized.

After reading and normalizing the signals, the controls and their respective treatments were exported into Excel files. This resulted in a total of 798 microarrays (517 treated assays and 281 controls) comprising 73 cell lines from 17 different tissues treated with 26 different anti-glycolytic agents. An additional 1014 microarrays were also downloaded on only one study (666 treated assays and 348 controls), which included 59 cell lines from 11 tissues treated with either sorafenib or rapamycin at different drug doses and exposure durations. The log base 2 foldchange (FC) was then calculated for each treated chip, and all the FCs were lastly fitted into two matrices: the first consisted of 199 foldchange retrievals, the second 666. The matrices thus contain expression data in form of FCs.

### Consensus clustering

Consensus clustering was performed using the *cola* package (version 1.8.1)^42^. This data classification technic was, foremost, done on the 199 experiments found on the GEO, to find a common denominator function for all glucose-challenged cells. Clustering was also performed on specific treatments, namely rapamycin and sorafenib from the 1014-sample accession, to study the effect of sample classification, while holding the treatment constant. Before performing the consensus partitioning, an important step was to clean up and perform data imputation to the input matrix. The *adjust_matrix()* function provided by *cola was hence used.*

After cleaning, quantile normalization was performed on each sample. This scaling method was used as it demonstrated better results compared to other scaling methods. For dimensionality reduction, the top 5000 features were first selected by the ATC top-value method (or "Ability To Correlate to other rows"). Then, the matrix was scaled by the selected rows and randomly sampled; these samples were partitioned by the skmeans (or "Spherical k-means") clustering method, a variant of the standard k-means clustering. Finally, the sampling and partitioning process was repeated 50 times to obtain a list of partitions.

The best-fitting number "k" of subgroups was evaluated using the average silhouette score, PAC score, concordance, and Jaccard index. The PAC score measures the proportion of ambiguous samples; 1-PAC thus represents the unambiguous sub-grouped samples. The best number of subgroups was the subgroup with the largest k having a product of the 3 scores greater than 0.9. The chi-square test was used to investigate the null hypothesis of whether the sample frequencies are equally distributed among the partitioned clusters.

### Functional analysis

The “signature genes” were then identified using the F-test for differential analysis. Signatures are simply the rows that show statistically significant specificity in one or more subgroups. These were further grouped by patterns among subgroups using k-means clustering and the appropriate number of signature groups was automatically selected. Functional enrichment was applied to each group of signatures separately. Gene ontology (GO) enrichment analysis was performed by applying the R package *ClusterProfiler*^43^ with *cola*’s function *functional_enrichment()*. GO terms were then clustered and visualized as heatmaps by the R package *simplifyEnrichment*^44^.

## Supplementary Information

Supplementary Legends.

Supplementary Table S1.

Supplementary Table S2.

Supplementary Table S3.

Supplementary Table S4.

## Data Availability

The generated datasets were downloaded for review (Supplemental Tables S3 and S4). The raw datasets analyzed in this study are available in the GEO repository, and all links to the accessions are given in Supplementary Table 1.
