# Supplementary material for: Enhancing cancer treatment and understanding through clustering of gene responses to categorical stressors
Source: Sci Rep. 2023 Apr 21;13:6517. doi: 10.1038/s41598-023-33785-w (PMC10121664; doi:10.1038/s41598-023-33785-w)
Supplement: Supplementary file 1 — Supplementary Legends. [file 41598_2023_33785_MOESM1_ESM.docx]

**Supplementary Information**

**Supplementary Table S1.** Samples accessions.

**Supplementary Table S2.** 195-sample classification.

**Supplementary Table S3.** 195-sample raw data.

**Supplementary Table S4.** 666-sample raw data.
